# Madness or sadness? Local concepts of mental illness in four conflict-affected African communities

**DOI:** 10.1186/1752-1505-7-3

**Published:** 2013-02-18

**Authors:** Peter Ventevogel, Mark Jordans, Ria Reis, Joop de Jong

**Affiliations:** 1 Department of Research and Development, HealthNet TPO, Amsterdam, the Netherlands; 2 War Trauma Foundation, Diemen, the Netherlands; 3 London School of Hygiene and Tropical Medicine, Center for Global Mental Health, London, UK; 4 Amsterdam Institute for Social Science Research, University of Amsterdam, Amsterdam, the Netherlands; 5 Leiden University Medical Center, Leiden, the Netherlands; 6 Department of Psychiatry, Boston University School of Medicine, Boston, MA, USA; 7 Rhodes University, Grahamstown, South Africa

**Keywords:** Burundi, Democratic Republic of Congo, South Sudan, Rapid assessment, Local concepts, Mental disorder, Idioms of distress

## Abstract

**Background:**

Concepts of ‘what constitutes mental illness’, the presumed aetiology and preferred treatment options, vary considerably from one cultural context to another. Knowledge and understanding of these local conceptualisations is essential to inform public mental health programming and policy.

**Methods:**

Participants from four locations in Burundi, South Sudan and the Democratic Republic of the Congo, were invited to describe ‘problems they knew of that related to thinking, feeling and behaviour?’ Data were collected over 31 focus groups discussions (251 participants) and key informant interviews with traditional healers and health workers.

**Results:**

While remarkable similarities occurred across all settings, there were also striking differences. In all areas, participants were able to describe localized syndromes characterized by severe behavioural and cognitive disturbances with considerable resemblance to psychotic disorders. Additionally, respondents throughout all settings described local syndromes that included sadness and social withdrawal as core features. These syndromes had some similarities with nonpsychotic mental disorders, such as major depression or anxiety disorders, but also differed significantly. Aetiological concepts varied a great deal within each setting, and attributed causes varied from supernatural to psychosocial and natural. Local syndromes resembling psychotic disorders were seen as an abnormality in need of treatment, although people did not really know where to go. Local syndromes resembling nonpsychotic mental disorders were not regarded as a ‘medical’ disorder, and were therefore also not seen as a condition for which help should be sought within the biomedical health-care system. Rather, such conditions were expected to improve through social and emotional support from relatives, traditional healers and community members.

**Conclusions:**

Local conceptualizations have significant implications for the planning of mental-health interventions in resource-poor settings recovering from conflict. Treatment options for people suffering from severe mental disorders should be made available to people, preferably within general health care facilities. For people suffering from local syndromes characterized by loss or sadness, the primary aim for public mental health interventions would be to empower existing social support systems already in place at local levels, and to strengthen social cohesion and self-help within communities.

## Background

Understanding local concepts of mental illness, and the related health care-seeking behaviour, is essential for the development of effective public mental health interventions after conflicts [1]. Elucidating popular nosologies of mental disorders not only can help health workers to better understand their patients, it can also prevent the imposition of categories that are meaningless to the patient and his social environment. This is important when health planners wish to address mental problems in non-Western cultural settings, such as in Sub-Saharan Africa, where formalized mental-health care is often limited to hospital-based services in major urban areas and where existing resources are insufficient, both in terms of human resources as well as in terms of coverage [2]. Conflict-ridden areas, in particular, are often devoid of mental-health professionals, while the mental health needs are huge [3,4]. Therefore, before starting an intervention programme to address mental health problems within a postconflict context, it is essential to know what local people think and which local concepts of mental distress they use. Such data may help to plan services that ‘make sense’ to potential users, including the way the services are organized and which problems they primarily should address.

Several challenges arise when studying ‘local concepts’ of mental illnesses. Firstly, what is a *local concept*? Local knowledge is continually reproduced and evolving [5] and is often somewhat idiosyncratic, and context dependent [6]. It may also vary due to historical changes, as well as shifting geographical boundaries. Past attempts to discover ‘folk illnesses’, described as ‘syndromes from which members of a particular group claim to suffer and for which their culture provides aetiology, diagnosis, preventative measures and regiments of healing’ [7], or ‘culturally bound syndromes’, described as the ‘clinical manifestation found in particular societies or cultural areas’ [8], have been criticized for their tendency to force local knowledge into a rigid system. Local medical knowledge may not be bounded by such a ‘system’, and local concepts may be ambiguous. What makes the study of local concepts particularly difficult is that they are, given the changing nature of our social world (including beliefs and culture) and the efforts of individuals to adapt to these changes, best viewed as an ongoing process or ‘work in progress’ [9].

A second challenge is how to define ‘mental illness’? The boundaries of what constitutes mental illness are influenced by cultural and other contextual factors and change over time [10]. Indigenous African categories of misfortune may not consider mental illness a separate, or distinct, category from other ‘nonmedical’ forms of misfortune, such as marital problems, failure to prosper or poor performance at school [11]. One classic challenge of crosscultural, psychiatric research is the need to avoid being blinkered by a rigid set of professional definitions of mental disorder that may have limited validity in different populations. This is known as the ‘category fallacy’, and was described by Kleinman [12]. Another challenge is to collect data that may have more than only local meaning and go beyond the specific boundaries of geographic location and historical context, and thereby may be useful for meaningful comparisons. This dilemma, often presented as an emic/etic dichotomy, manifests itself from the very beginning of the research: how do we define the subject, mental disorders, under investigation? Do we take the professional classifications as our starting point and check their ‘fit’ within each context? Or do we start with the local definitions, using ethnographic methods to elaborate local conceptualizations, while at the same time acknowledging the inherent implication that the boundaries of ‘mental illness’ may be fluid?

This paper presents the results of a rapid ethnographic assessment to explore local concepts of mental disorders in four settings in Africa. As a working definition for ‘mental disorder’, the authors used the description by the World Health Organization, referring to ‘disorders or problems characterized by symptoms expressed in abnormal thoughts, emotions, behaviour and relationships with others’ [13]. The boundaries of a mental disorder were not further specified, in order to give respondents the opportunity to describe the local concepts and local syndromes they found useful and appropriate. We define a local syndrome as ‘a widely recognized prototypical ailment that encompasses a fuzzy set of associations coalescing around one or more core cultural symbols’ (cf. Nichter [9]).

The assessment aimed to generate data to assist HealthNet TPO (an international nongovernmental organization involved in health care development in postconflict settings) to integrate mental health activities into existing public health programmes in South Sudan, the Democratic Republic of the Congo (DRC) and Burundi. Information on local names for mental problems was gathered. Additionally, what respondents saw as defining features and causes related to these conditions and what they commonly did to address these problems were also discussed.

## Methods

### Study settings

The study was conducted in four African settings, where HealthNet TPO implements programmes to construct or reconstruct health care systems. The fieldwork was done between March and October of 2007.

#### Setting 1: Kwajena Payam (South Sudan)

Kwajena Payam is an administrative district in Western Bahr el Ghazal State. The main ethnic group are the Jo-Luo, one of the smaller ethnic groups in South Sudan. They speak a form of the Nilotic language *Luo* (*dho-luo*) and are culturally and linguistically related to other *Luo*-speaking people, such as the Shilluk in Sudan and the Acholi in Uganda. During the second Sudanese civil war (1991–2005) Kwajena Payam, one of the main settlements of the Jo-Luo, saw a temporary influx of other ethnic groups, mostly Dinka [14]. Most people live in *tukuls* (round huts) constructed with mud, branches and thatched roofs, although some reside in tents provided by aid organizations. The land is fertile, bushy forest. The population of Jo-Luo are agriculturalists, mainly growing beans and sorghum, but many of them, following their Dinka neighbours, have also become cattle keepers. In Kwajena Payam, there are few health facilities and no formal mental health services. The closest town is the state capital Wau, around 100 km from the district (three hours’ drive in the dry season). Hospitals in Wau do not have facilities to treat people with mental disorders.

#### Setting 2: Yei (South Sudan)

Yei River County is one of the most southern administrative units of South Sudan. It has direct road connections to the DRC and Uganda. The county is culturally dominated by the Kakwa, who live in the borderlands between Uganda, the DRC and South Sudan. The Kakwa survive on a mix of agriculture, pastoralism and increasingly from trade with neighbouring countries. They speak *Kakwa*, one of the Nilotic Bari-languages of South Sudan. During the second Sudanese civil war it was a stronghold of the Southern Sudanese rebel movement. The people are relatively well educated, with many returning refugees having received basic education in Uganda. At the time of this research, there were no formal mental health services in Yei River County. The county is approximately 100 km from the capital of South Sudan, Juba, which has a neuropsychiatry unit at the teaching hospital (but no psychiatrist and no qualified psychiatric nurse). The few patients in Yei River County who could afford transportation and treatment costs are often sent to treatment facilities in northern Uganda.

#### Setting 3: Butembo (DRC)

Butembo is situated in the northern part of the North Kivu Province in the DRC. This area has experienced decades of political and ethnic violence as a result of two wars (1996–1997) and 1998–2003) and the influx of refugees from Rwanda [15]. The area around Butembo is the homeland of the Wanande, numbering about one million people. They speak *Kinande*, a Bantu language. Economically, the region around Butembo is dependent on subsistence agriculture and trade. The only psychiatric institution in the area is a small facility in Butembo town, headed by a psychiatric nurse who trained in the 1980s. The closest psychiatric hospital is in Goma, the capital of North Kivu; however this facility is too far away to be of any use to the residents of Butembo.

#### Setting 4: Kibuye (Burundi)

Kibuye is a district in central Burundi, a country that has experienced cyclic outbreaks of ethnic violence, notably in the 1970s and 1990s [16]. Fighting between the Tutsi-dominated national army and rebel groups from the Hutu majority killed 300,000 people and displaced over one million between 1993 and 2003 [17]. The conflict caused the destruction of socioeconomic infrastructure countrywide. There are no formal mental health services in the district, but in the provincial capital Gitega, approximately 50 km away, a monthly mobile mental health clinic is run by the provincial hospital [18].

### Procedure

The authors used the methods of rapid ethnographic assessment with qualitative research techniques to collect data within a short period for programme development [19]. In each area, two or three research assistants were selected. Their educational background varied from secondary school leavers (Sudan) to BSc psychologists (DRC and Burundi). They were fluent in the local language of the participants, and in English or French. The first author trained the research assistants in each setting, during a three-day training that consisted of preparing and using the instruments and methods. The study protocol was reviewed and approved by the Research and Development Department of the concerned NGO (HealthNet TPO). This included a review of procedures and ethics. The research plan was also discussed in all four settings with the local health authorities who gave approval. The objectives of the study were read out to all participants and verbal consent was obtained before the interviews and focus group discussions.

#### Focus groups discussions (FGDs)

FGDs were held in a public venue such as a school, a local health care centre or a church. Discussions followed a topic guide around one question: ‘We would like to talk with you about problems and illnesses that manifest through problems in thinking, feeling or behaving*.*’ The participants were asked to describe how such problems or illnesses manifested, what causes were related to them and how such problems or illness were usually managed. The duration of each FGD was between one and a half and three and a half hours.

The first FGDs were conducted with the first author as co-facilitator and were passed over to the research assistants when they were able to do the FGDs without supervision. Participants for the FGDs were purposely selected, by the research assistants and community leaders, on the basis of gender and age. The aim, as explained to the community leaders, was to have a broad representation of people in the community. First-degree family members of each other were not allowed to participate in the same group.

Separate groups were held with men and with women, with older persons and with youngsters (aged 16–25, the defining criterion being ‘unmarried’). In total, 31 FGDs (with a total of 251 participants, about half of whom were female) were organized. See Table 1 for demographic details of the participants. A minimum of six focus groups per location was planned. However, in two locations (Butembo and Kwajena Payam), no data saturation was reached after six focus groups; therefore, additional focus groups were conducted.

**Table 1:** Demographic characteristic of participants in focus group discussions

	**Butembo (DRC)**	**Kwajena (South Sudan)**	**Yei (South Sudan)**	**Kibuye (Burundi)**	**Total**
Number of groups	9	10	6	6	31
Rural	4	10	5	6	25
Urban	5	0	1	0	6
Gender of participants					
Male (% of total)	32 (43%)	40 (48%)	27 (60%)	24 (50%)	123 (49.0%)
Female (% of total)	42 (57%)	44 (52%)	18 (40%)	24 (50%)	128 (51.0%)
Mean age (years) of participants in subgroups					
Youngsters	18.7 (16–20)	20.4 (15–27)	25.5 (22–29)	20.0 (17–23)	
Men	37.3 (24–52)	46.2 (32–51)	48 (30–61)	45.2(31–60)	
Women	40.8 (32–39)	37.5 (31–51)	38.3 (27–60)	36.0 (23–46)	
Elder men	49.0 (44–67)	50.1 (30–70)	46.4 (40–62)	54.8 (39–70)	
Elder women	55.8 (42–76)	43 (37–50)	-	44.3 30-63	
Education					
Mean number of years in school (spread)	4.9 (0–13)	1.6 (0–10)	4.5 (0–12)	3.7 (0–9)	
Languages used	Kinande	Kakwa	Luo	Kirundi	

#### Key informant interviews (KII)

During meetings with the community leaders and during focus groups, a broad category of people identified as ‘experts on mental problems’ were approached for semistructured interviews. The first author and research staff conducted the interviews with key informants, such as traditional and religious healers, and health-care staff. In each setting, two or three traditional healers were interviewed and between three to seven other key informants (general health workers, policy-makers). In total, twenty-six key informants were interviewed. They were asked the same questions as the participants of the focus groups, but in addition were asked more in detail about their own work.

### Data analysis

The discussions, including the questions asked by the facilitator, were noted by one of the research assistants in the local language and later translated into English. During this translation process, the two research assistants checked the work of the other and, when required, assisted each other in modifying the translations. The information collected from the focus groups was reviewed by the authors using content analysis, with an iterative coding procedure [20]. Only items that were mentioned in two or more focus groups in one setting were included in the analysis. For each local category, responses were ordered according to ‘symptoms’, ‘causes’ and ‘treatment options’ [21,22]. These data were analyzed by the first author, and emerging themes and categories of illness were discussed with the research assistants. The resultant adaptations were made on a consensus basis.

## Results

A brief overview of the local syndromes found in each setting is presented first. Also, for the purposes of this paper, syndromes that referred to epilepsy-like syndromes, mental retardation and drug and alcohol conditions were excluded, although they were mentioned in all settings. These will be discussed in separate papers.

### Local syndromes in Kwajena (South Sudan)

#### Moul

Respondents describe people with *moul* as aggressive (‘fighting with people, throwing spears or setting houses on fire’) with bizarre behaviour, such as: ‘walking around naked, eating faeces or collecting rubbish’:

‘They say things that make no sense. They talk about one thing and in the next sentence they talk about something completely different. So a normal person cannot understand them.’ (Man in FGD, 12 April 12 2007)

#### Wehie arenjo / wehie arir

*Wehie arenjo* (*‘*destroyed mind’) or *wehie arir* (‘disturbed mind’) refer to those who used to be normal, but suddenly behave abnormally. Features mentioned by the respondents include: ‘becoming very sad’, suicidal thoughts and display strange behaviours, such as ‘talking or laughing when no one is around’. People with this condition are thought to be easily angered and aggressive. It is also thought to be a temporary, albeit reversible, condition:

‘He behaves like someone drunk. The next morning he realises what he has done and then regrets his behaviour. *Wehie arenjo* is less severe than *moul*, because *wehie arenjo* can return to normal.’ (Man in FGD, 10 April 10 2007)

#### Nger yec

People with *nger yec* (‘*cramped stomach*’) are believed to always be sad. They have little appetite, are inactive and do not work. Many have suicidal thoughts. They cannot quieten their minds and often sleep only a few hours a day. A person with *nger yec* feels weak and tired and often believes that his or her situation is hopeless. Frequently, this is accompanied by diarrhoea, which is often green in colour and can sometimes cause collapse due to weakness. They are forgetful and tend to isolate themselves:

‘If you tell him something, he will forget it. When people talk to him, he does not listen, because his mind is somewhere else. They do not walk to their neighbours, but hide in their houses and will not come to a meeting like the one we are having now.’ (Woman in FGD, 10 April 10 2007)

### Local syndromes in Yei (South Sudan)

#### Mamali

The main feature of *mamali (*‘disturbed mind’) is aggressive behaviour, such as ‘throwing stones at people’. Other characteristics are: ‘talking when no one is present’, bizarre behaviour including eating dirty or inedible things, ‘walking naked’, bad hygiene and self-neglect, social isolation and speaking in an unintelligible manner.

#### Ngengere

A specific type of *mamali* is called *ngengere*. This is an acute condition characterized by: aggressive behaviour (fighting, throwing stones and shouting), disturbed speech (singing songs all the time), emotional instability (‘they change in a moment from laughing to crying’) and running away, into the bush.

#### Yeyeesi

*Yeyeesi* (‘many thoughts’) is used to indicate people ‘whose mind is always busy with thoughts’. People with *yeyeesi* often isolate themselves, lack appetite, feel sad and often cry. Usually they cannot sleep properly and sometimes have suicidal thoughts:

‘Such a person thinks everything in the world is very bad. When something good happens, for example when he gets a present, he will only be happy for a short time and then be sad again.’ (Man in FGD, 30 March 2007)

Other characteristics mentioned included: absent-mindedness, frequent headaches, self-neglect and poor hygiene, and irritability.

### Local syndromes in Butembo (DRC)

#### Erisire

A *musire* (a person with *erisire)* is typically thought to be verbally and physically aggressive (throwing stones and beating people). The behaviour of *musire* is uncoordinated*,* ‘without order in their actions’*,* as indicated by taking their clothes off, walking naked, eating inedible things (like leaves from the street), walking aimlessly and sitting down in dirty places. People with *erisire* talk about things that are not relevant or are unable to logically follow the course of a discussion:

*‘*One can understand the words they are saying, but these are only things that are not relevant. They say whatever comes into their mind and they say it whenever they like.’ (Man in FGD, 8 March 2007)

Other symptoms include: ‘singing songs all the time’, ‘laughing or crying at inappropriate moments’, ‘talking to people when no one is there’, ‘stealing things’ and ‘not realizing they are mentally ill’. A specific type is *erisire ry’emumu* (silent *erisire*), which was described in two focus groups and is characterized by social isolation, not speaking, absence of movement and a profound sadness. In two urban groups, another type of *erisire* was described, characterized by too much activity, talking, dancing and singing excessively and an inappropriate, exalted mood.

#### Amutwe alluhire

*Amutwe alluhire (‘tired head’*) is used to indicate someone who is sad, irritable or nervous, and often cries without reason. A person with *alluhire* is ‘confused.’ They are easily angered or irritated, and feel neglected by family and friends. They are often forgetful and socially withdrawn. These problems become visible during social contact with others:

‘He has difficulties in contact with other people. He does not recognize people, because he is occupied with his thoughts. His mind is somewhere else.’ (Elderly woman in FGD, 7 March 2007)

### Local syndromes in Kibuye (Burundi)

#### Ibisazi

According to all respondents in Kibuye, the key features of people with *ibisazi* are aggression and lack of respect for others. They may have a mad or bewildered look in their eyes and exhibit bizarre behaviour, such as: going naked, collecting useless things and neglecting personal hygiene. Some talk all of the time, while others hardy speak at all.

#### Ibonge or akabonge

People with *ibonge* are either always talking and dwelling on what they have lost or are very withdrawn and hardly speak. Other features are social isolation, always feeling sad, not allowing anything to cheer them, sleep problems and suicidal thoughts. They exhibit no interest in anything. Other words that are used to indicate a state of sorrow, in which a person is not able to function normally, are *agahinda*, *kinemura* or *akarunga*. People suffering from *ibonge* often sing *gucurintimba* (melancholic songs), full of regret and sorrow, about the mistakes they have made and how everything was lost. The syndrome of *ibonge* can also include symptoms such as ‘having a deranged mind’ and ‘talking to oneself’. The neglect of social obligations is an element that was also stressed. Sometimes *ibonge* is distinguished from *kuyinga*, a more dangerous condition in which a person becomes a ‘quiet fool’. This condition is characterized by disorganized behaviour, such as gathering plants and rubbish, but also by a lack of aggression.

#### Ihahamuka

People with *ihahamuka* are highly fearful and startled by loud noises. They are always ‘on alert’, easily distracted by things within the environment and often silent. They may also sleep badly and have no appetite. *Ihamamuka* is always a reaction to traumatic events, for example: witnessing massacres during the war, rape or a bad car accident:

‘They are always alert, as if there is always danger, but this danger is not real. At night, while they are sleeping, they often awake suddenly. Then they cannot fall asleep again. They are also afraid to go anywhere.’ (Woman in FGD, 12 July 12 2007)

### Comparing syndromes

#### Symptoms

**Figure 1:**
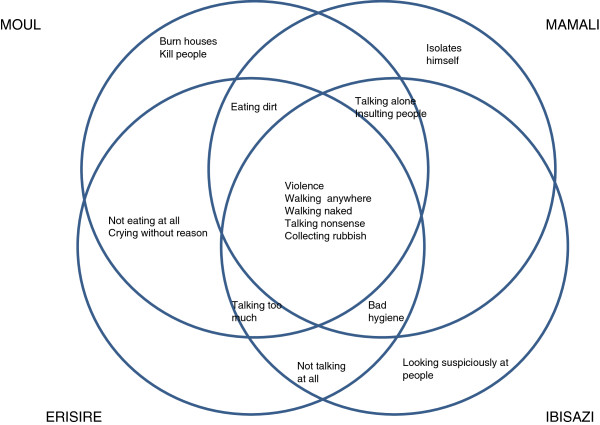
Local syndromes with behavioural disturbances and violence as common features in four African settings.

There are several similarities within the four settings. The local syndromes of *moul* (Kwajena, South Sudan), *mamali* (Yei, South Sudan), *erisire (*Butembo, DRC*)* and *ibisazi* (Burundi) would all have to be literally translated as ‘madness’ and are all ‘conditions related to severe behavioural disturbance’. Among the defining features are interpersonal violence, chaotic behaviour (walking aimlessly or naked, collecting rubbish, etc.) and ‘talking nonsense’. Other elements, such as talking when alone, talking too much, eating dirt and bad hygiene, were mentioned in three of the four locations as characteristic symptoms. This is visualized in Figure 1, in which each circle represents a local concept from one of the four research settings. Thus, symptoms that were mentioned as defining characteristics for all four conditions are situated in the centre of the picture, enclosed by all four circles. A symptom that was mentioned as a symptom for three of the four conditions is enclosed by three circles.

**Figure 2:**
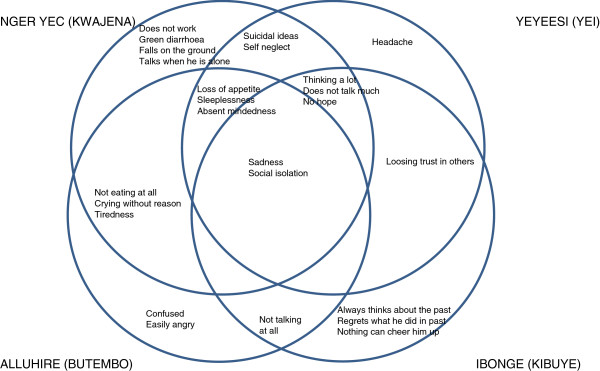
Local syndromes with sadness and social isolation as a common feature in four African settings.

The local syndromes of *nger yec (*Kwajena), *yeyeesi* (Yei), *alluhire* (Butembo) and *ibonge* (Kibuye) share some features such as feeling overwhelmingly sad and social withdrawal, but there are significant differences as well. The unique symptoms include *‘*green diarrhoea’ (*nger yec*), ‘headache’ (*yeyeesi*), ‘confusion’ and ‘irritability (*alluhire*), as well as self-remorse and dwelling on the past (*ibonge*). This is visualized in Figure 2, in which each circle represents a local concept from one of the four research settings. Thus, symptoms that were mentioned as defining characteristics for all four conditions are situated in the centre of the picture, enclosed by all four circles. A symptom that was mentioned as a symptom for three of the four conditions is enclosed by three circles. These local concepts are referred to as ‘conditions related to sadness and social withdrawal’.

#### Aetiology

In all four research settings, the locally described conditions are thought to be related to a wide range of potential aetiological factors. These are summarized in Table 2 and include supernatural, natural and psychosocial causes.

**Table 2:** Perceived aetiology of locally defined conditions in four African settings

	**Supernatural**	**Natural**	**Psychosocial**
**Kwajena (South Sudan)**			
*Moul*	Spirits of dead people (*cien*)	Malaria	Thinking too much
	Malevolent spirits (*djok, arop*)	Meningitis	Loss of properties and loved ones due to the war
	Violating a taboo		
	Being cursed		
*Wehie Arir*	‘Perhaps somebody is behind your misfortune’ (indicating sorcery or witchcraft)		Having lost children or property
*Nger yec*			Recent loss (of a person or property)
**Yei (South Sudan)**			
*Mamali*	Being bewitched	Cannabis	*Yeyeesi* (‘thinking too much’)
	Attack by spirits from water or forest (*a’bionga* or *dulako*)	Alcohol	‘Too many problems’
		Brain damage	Family disputes
		Typhoid fever	
		Born this way	
*Ngengere*	Being bewitched	Drugs	
		Alcohol	
*Yeyeesi*			Loss of beloved person
			Loss of property
			State of poverty
			Family disputes
**Butembo (DRC)**			
*Erisire*	Bad spirits (*virumu*)	Cerebral malaria	Death of a loved one
	Scorcery *(*by a *mukumu* – traditional healer)	Epilepsy	Being rejected in love
	Bad spell (*lirengo*)	Drugs	
		Alcohol	
*Alluhire*			Worrying about problems
			Poverty
			Family problems
			Death of loved ones
			Rape
**Kibuye (Burundi)**			
*Ibisazi*	Sorcery	Malaria	*Ibonge* (see below)
	Angry ancestor spirits	Fall on head in accident	Having lost belongings
	Bad spirits	Change in the blood	Seen too many bad things in the war
		Drugs	
		Alcohol	
*Ibonge*			Death of a loved one
			Loss of property
			Loss of livelihood
			Having witnessed atrocities during the war
			Worrying about bad health
*Ihahamuka*			Having witnessed terrible things in war
			Having been raped
			Car accident

#### Supernatural forces

In all four areas, respondents described supernatural forces as a causal factor for conditions related to severe behavioural disturbance. Local cosmologies within the four settings were not identical, however, with different ways of conceptualizing the supernatural realm. For example, it can be related to ‘bad spirits’ (from rivers, lakes or rocks), disturbed ancestral spirits, violating a taboo or being cursed or bewitched (although this is usually through the mediation of a sorcerer or a ‘bad’ traditional healer):

*‘*A person can get *mamali* when the person has stolen something from another who seeks spiritual revenge, or is attacked by spirits from the mountain, the waters or from the thick forests.’ (Man in FGD, Yei, South Sudan, 30 March 2007)

‘*Arop* (a malevolent spirit) can come into the house with, for example, a goat or a cow that you have bought, and bring the spirit with him. If you are not aware this spirit is in the house, it can start killing people or cause *moul*. You have to do something, like slaughtering an animal, so the *arop* knows that you respect him.’ (Woman in FGD, Kwajena, South Sudan, 12 April 2007)

Spiritual causes were never mentioned for conditions that have sadness and social withdrawal as common features.

#### Natural diseases

In all four settings, infectious diseases (e.g. malaria) were mentioned as potential causes of the conditions related to severe behavioural disturbance. Apart from in Kwajena (South Sudan), the use of alcohol and drugs were also mentioned as a cause, in all settings.

For ‘conditions related to sadness and social withdrawal’*,* natural diseases were not mentioned as a potential cause.

#### Loss and worry

All groups mentioned loss as main cause of ‘conditions related to sadness and social withdrawal’. This could have been the loss of livelihood and properties, but often involves the death of a loved one, particularly children. It could also be induced by living in miserable conditions, such as extreme poverty, being physically ill for a long time, by family problems such as divorce or too many responsibilities:

*‘*When you lose someone you loved very much, or when you lost a lot of money or when your house is burnt, this can cause illness. You can get really sick from it, but when you go to the health centre, the doctors cannot find any disease. […]When a father dies and he has three sons, all will cry. But one son cries too much. That one has *nger yec.* He feels it in his stomach. Sometimes a person can even tie his belly with a rope to stop the cramp.’ (FGD, Kwajena, South Sudan, 10 April 2007)

‘*Akabonge* is seen especially with adult people who have lost their children and goods. They continue to mourn and despair completely. People with *akabonge* remain silent, as if they are dumb. They are absent-minded and are not interested in life.’ (FGD, Kibuye, Burundi, 13 July 2007)

The cause of *ihahamuka* (Kibuye, Burundi) is related to having witnessed gruesome events, for example massacres during the war, or surviving extreme events, such as rape or a bad car accident.

In all four settings, people also made a link between situations of severe loss and conditions related to severe behavioural distance and violence. In Butembo, for example, respondents mentioned that *erisire* may follow a major setback in life, such as the loss of a beloved family member or rejection by someone they love. Respondents, within different settings, used similar explanations:

‘*Mamali* can be caused through *yeyeesi*, for example when one has lost all his properties or dear ones.’ (KII with healer, Yei, South Sudan, 30 March 2007 )

This is in contrast to the acute forms of severe behavioural disturbance and violence that were distinguished in Yei and in Kwajena. These were related to clearly identifiable factors. *Wehie arenjo*, the acute form of behavioural disturbance that was described in Kwajena (South Sudan), was overwhelmingly thought to be related to loss, which leads to *par keter* (‘thinking too much’), which in turn could lead to *wehie arenjo:*

‘Those people think too much. For example, when many of your children have died, then a person can become very sad and think too much. They think in a negative way.’ (KII with healer, Kwajena, South Sudan, 30 April 2007)

Across all geographic locations, respondents reported that sadness and social withdrawal could contribute to conditions related to severe behavioural disturbances; in Burundi *ibonge* can, for example, lead to *ibisazi*. For example, someone who had lost all their belongings, or was haunted by memories of the war, would first develop *ibonge* or *ihahamuka*, but could eventually reach a stage of *ibisazi*.

#### Treatment

In all four settings, treatment decisions were strongly dependent on the perceived cause of the condition, which was not always apparent from the *‘*symptoms’. What seemed to have similar effects could have very different causes. For example, if a condition was related to a natural cause, such as a disease with fever, health facilities were mentioned as a treatment option. Usually, however, the causes of conditions related to severe behavioural disturbance and violence were not immediately clear. Therefore, a first step in the help-seeking process would be to discover the cause, and in particular, to resolve it if supernatural factors were believed to be present.

Perceived treatment options for locally defined conditions in four African settings are given in Table 3.

**Table 3:** Perceived treatment options for locally defined conditions in four African settings

	**Traditional healers**	**Health care facilities**	**Family and community interventions**
**Kwajena (South Sudan)**			
*Moul*	Visit *ruedbedho* (‘spear master’) to chase spirits away	Health centre in case malaria is cause	
	Ngadeyeadh (herbalist) who can give herbs		
*Wehie Arir*	Visit *ruodbedho* (‘spear master’) to understand the cause	Health centre (medicine to calm patient down)	Try to replace the things or persons he has lost
		Medicines if caused by malaria	Relatives or elders in community should talk to person
			Pray in the church together with the patient
			Prevent person from drinking alcohol and smoking cannabis
*Nger yec*			Relatives or elders in community should talk to person and give advice to overcome the sadness
			Compensate person for the losses he suffered
			Invite the person to come to your house
**Yei (South Sudan)**			
*Mamali*	Visit a *buni* (traditional soothsayer) to find out the cause and perform rituals to chase away the spiritual forces		Praying to calm down a patient
*Ngengere*	Some healers have herbs to calm person down		
*Yeyeesi*			Family or religious leader talk with the person and give him advice
			The family and neighbours should also help the patient not to be alone and to involve him in activities, in particular those that can give him income
			Elders from the church can visit the person and pray together.
**Butembo (DRC)**			
*Erisire*	*Mukumu* (traditional healers who work with spirits), if there are supernatural causes	Visit a health facility to check if there is malaria or another physical cause	
	*Musaki* (herbal healers) for herbal medicine	Visit mental health centre in town	
	Christian pastors who can pray with the patient		
	In case of possession by ancestral spirits, one should construct a *vuhima*, a small house for the ancestors		
*Alluhire*			Provide him money, goods or work
			Visit the person
			Pray with the person
			Ensure that the person is not alone.
			Involve him in communal work in the village
**Kibuye (Burundi)**			
*Ibisazi*	In case of sorcery: traditional healer	Provincial hospital	
	In case of bad spirits: praying and rituals in the church (three groups)		
*Ibonge*			Family and try to comfort person
			Encourage person to talk about his problems
			Replace the loss
*Ihahamuka*			Family to listen to the person
			Family and friends should help the person to do the things he is afraid of

Participants, in all settings, were very pessimistic about treatment options for conditions related to severe behavioural disturbances:

‘It is difficult to help because the problem is inside the mind of the person. There are no medicines for this.’ (KII with community leader, Yei, South Sudan, 31 March 2007)

‘We bring a person with *moul* to a *ruodbedho* (spear master) who can chase bad spirits away, or can do away with sorcery done by another spear master. We also go to *ngadyeadh* (herbalist) who can give medicine, but often this does not help.’ (Elderly man in FGD in Kwajena, South Sudan, 9 April 2007)

If a spiritual cause is established, rituals can be done to banish harmful spiritual forces:

‘There is not a single treatment for *ibisazi*. In the case of sorcery, one should go to a traditional healer. In the case of bad spirits, one has to chase the spirit away by praying and rituals in the church, and in cases that do not have a clear supernatural cause, one should go to the hospital. If you do nothing, the patient will not improve and will finally die.’ (Elderly man in FGD, Kibuye, Burundi, 13 July 2007)

In one setting (Yei, South Sudan), traditional healers made the distinction between the chronic condition *mamali* (considered very difficult to treat) and the more acute condition *ngengere* (easy to treat). They believe that herbs for *ngengere* will calm the patient and, with use, the problem will disappear completely.

In Butembo (DRC), participants in all groups reported that patients with *erisire* can be treated in the mental health centre in the town. In the rural areas, people mentioned that the distance to the mental health centre was a problem. Butembo was the only place where people mentioned psychiatric treatment, and indeed was the only setting with such a facility available. People with *erisise* are thought to improve through treatment with Western medication, but not when sorcery or spirits caused the condition. In these cases, a visit to *mukumu* (traditional healers who work with spirits) is recommended.

Respondents in all settings believed that *‘*conditions related to sadness and social withdrawal’ were not diseases, but states caused by circumstances. Therefore, health centres and traditional healers were not thought to be effective in helping those with such conditions. For these conditions, in all four settings, the actions required were believed to be social and involved a combination of advice, comfort, practical support and breaking through social withdrawal.

‘The first thing to do to help a person with *nger yec* is to talk to him, and to give advice to overcome his sadness. Elders in the community, or relatives, can talk with him and tell him to be courageous, and about other people who have been in the same situation and survived.’ (Man in FGD, Kwajena, South Sudan, 10 April 2007)

‘When a mother has lost many children and gets *ibonge*, a family member can replace the lost children by sending one of his own children to live with her, and can help her.’ (Woman in FDG, Kibuye, Burundi, 12 July 2007)

A person suffering from *alluhire* can be helped by providing material assistance, work or a good house. It can also help to seek distraction, so he will not always think about the bad things, for example by visiting the person. The family of the person should be advised how they can help. Praying with the person can also help. With good assistance, a person with *alluhire* will become normal.’ (Woman in FGD Butembo, DRC, 7 March 2007)

## Discussion

The group of local syndromes defined by severe behavioural disturbances have considerable similarities with ‘psychotic disorders’ (including manic states). Local syndromes that were characterized by sadness and social withdrawal have similarities to what used to be known collectively as ‘neurotic disorders’. However, in the current international psychiatric classifications such as DSM V and ICD-10, these would be characterized as mood disorders, complicated bereavement and/or anxiety disorders.

### Conditions related to severe behavioural disturbances

The concept of ‘psychosis’ in psychiatry encompasses five elements: confused thinking, false beliefs, hallucinations, changed emotions and disturbed behaviour. The local concepts identified in this research do not emphasize all these symptoms, but tend to focus on ‘behavioural problems’, particularly violent and chaotic behaviour, and ‘cognitive symptoms’ (‘speaking in a way that people cannot understand’ or ‘saying things that are not real’). Emotional expressions, such as crying and laughing without reason, were found, but these were not considered as typical for severe mental disorders. Hallucinations were not mentioned in any of the settings; however, in all four settings, examples were given of behaviour that could indicate auditory hallucinations, such as ‘speaking when there is no one around’*.*

Perhaps some elements of popular discourse around psychotic disorders in this study bear witness to prevailing norms and ideals within a particular society. For example, the Luo from Kwajena in South Sudan revere male strength, and see men primarily as warriors. Men are often armed with spears. In their descriptions of *moul* and *wehie arir*, they emphasized violent aspects, such as killing people and burning houses. The Burundians in Kibuye stressed the ‘disrespectful behaviour’ of those with mental disorders, which may reflect the importance of harmony and modest, respectful behaviour within their society (as conferred by the local concept of *indero*). Public displays of emotion are frowned on in Burundi.

Lay descriptions of psychotic disorders in Sub-Saharan Africa emphasize behavioural disturbance and disruption of social norms, yet do not often contain symptoms related to thought disturbance and perceptual symptoms [23-25]. The list of characteristics of people with ‘psychosis’, reported by respondents from four East African ethnic groups in a classical study by Edgerton [26], included: walking naked, being violent, arson, and talking nonsense. The concepts of *moul*, *mamali*, *erisire* and *ibisazi* in our study are quite similar and significantly overlap with the psychiatric concept of psychosis. They are, however, less narrowly defined and include categories that in current psychiatric nosology are often separated from psychosis, such as manic episodes and delirium.

The Luo and the Kakwa respondents used separate names for acute, and potentially time limited, states of severe disturbance (*wehie arenjo* in Kwajena and *ngengere* in Butembo), with pathology centred on problems with interpersonal behaviour. Professional psychiatric classifications would refer to these acute syndromes as brief reactive psychosis, acute mania, non-affective acute remitting psychosis, ‘bouffée delirante’ or early-stage schizophrenia. Similar distinctions between chronic states of psychosis, and between acute forms characterized by aggression and behavioural disturbance, have been described in other African societies [26-29].

### Local aetiologies for conditions related to severe behavioural disturbances

The respondents in this study list a wide range of possible causes for disorders with severe behavioural disturbances, including spiritual, natural and psychosocial factors. In the literature on African causal theories for mental disorders, the role of spiritual aetiology is often emphasized. Indigenous healers in Uganda indicated that the cause of these disorders was not specific to the person, but could be due to any family member or members neglecting cultural practice [30]. However, not all cases of psychotic disorders are attributed to supernatural forces. Edgerton [26] found that psychosis is not always attributed to witchcraft, and it was often regarded as an illness occurring for no reason or as the ‘natural result of life stress’. In the literature on local aetiological beliefs with regards to psychotic disorders in Africa, a wide range of factors have been described, such as substance misuse [31], nutritional factors [32], diseases of the blood [33], malaria [24] and *‘*worms in the brain’ [26] In this study, most of these factors were mentioned by the respondents, but there was variance between the settings. Among the Luo (arguably the setting that has been least influenced by monotheistic religions), spiritual causes were more prominent. The Kakwa also mentioned spiritual causes, but more frequently mentioned natural causes (cannabis and alcohol use) and psychosocial causes (‘too much thinking’).

### Treatment

In three of the four settings, respondents indicated that they thought severe mental disorders could not be effectively treated by either traditional healers or in biomedical health facilities. They generally do not seek help in modern health facilities because they are not aware that medications to treat psychotic symptoms may exist. This is quite understandable because, in three of the four locations, there were no health workers who were trained in the diagnosis or management of mental disorders and no psychotropic medication was available in the health facilities. The exception is Butembo, where treatment by Western medication is generally thought to be effective; this may be due to the long-term presence of an active, and highly respected, psychiatric nurse. Respondents were more optimistic about treatment options for acute psychotic conditions. This may be an indication that when psychiatric treatment options for severe mental disorder are made available, people will try them out and continue using them once they experience positive effects.

### Conditions related to sadness and social withdrawal

In various African populations, conditions can be found that are assigned to ‘too much worrying’ or ‘too much thinking’ [34-37]. To what extent are these local concepts identical to psychiatric concepts for affective disorder, such as depressive disorders? On first sight, the resemblance is striking. For example, the Luo description of *nger yec* includes all symptoms of the DSM-IV definition of depression, with the exception of excessive or inappropriate guilt. However, the defining feature cited by the Luo respondents was not the emotional features, but the existence of typical somatic symptoms, in particular pressure on the stomach and diarrhoea.

*Ibonge* in Burundi also resembles, but is not identical to, depression. *Ibonge* signifies ‘sadness resulting from a multiple sufferings’ and *kurwara akabonge* is ‘being sick of sadness’ [38]. In Rwanda, which shares many linguistic and sociocultural features with Burundi, similar local categories were identified. These included *agahinda gakabije* (with symptoms such as deep sadness, isolation, lack of self-care, loss of mind, not able to work, feeling life is meaningless, not pleased by anything and difficulty in interacting with others) [39]. The features of *ibonge* and *agahinda* may seem quite similar to Western concepts of depression, but also reflect a transgression of what is considered ‘good behaviour’. For example, the emphasis that Burundian culture places on harmony and not showing emotions to others.

The conditions identified in this research are not discrete diagnostic categories with a specific set of symptoms, but have fluid boundaries and are applied pragmatically. For example, while the Nande concept *alluhire* may be associated with features of major depression, it is also a rather idiomatic expression to communicate that a person does ‘not feel well’ and is overwhelmed by the tasks of life. *Alluhire* should thus not only be understood as a local syndrome, but also as an ‘idiom of distress’: a culturally prescribed way of communicating distress. An idiom of distress may be indicative of psychopathological states that undermine the well-being of a person, but may in other cases better be seen as adaptive reactions to a situation of distress, and thus be a way of coping with distress [9,40].

### Local aetiologies for condition related to sadness and social withdrawal

The local concepts related to sadness and social withdrawal in this assessment are thought to be the consequence of identifiable contextual factors, such as severe loss or adversity that, once removed, will result in improvement. Personality factors (such as being ‘weak’) play a role as well. As elsewhere in Africa, these conditions are less likely to be seen as a medical or mental disorder, but are more likely to be ascribed to social or spiritual problems with poverty, social issues, major life events and ‘thinking too much’ [23,41-43].

### Treatment

Despite diversity in the symptomatic descriptions, management of conditions related to sadness and social withdrawal is quite similar, especially as these conditions are not seen as medical disorders and therefore treatment is rarely sought in modern health care facilities. People believe the management should be entirely psychosocial and aimed at improving the economic situation, increasing social support and decreasing social isolation and loneliness.

### Psychotrauma

Local African concepts of mental conditions related to ‘traumatic events’ vary considerably from the DSM concept of posttraumatic stress disorder, as demonstrated in Gambia [35], Rwanda [39] and among Darfuri refugees in Chad [44]. The latter group distinguished two differing local concepts. The first, *hozun*, had similarities with depression and some elements of posttraumatic stress disorder. The second, *majnun* (literally ‘madness’) also contains some posttraumatic stress symptoms similar to major depression, but in general the syndrome is defined by psychotic symptoms mentioned by the Darfuri respondents (such as ‘talking when you are alone’, ‘talking in a way others cannot understand’ and ‘doing things others consider foolish’). In fact, local categories of *hozun* and *majnun* would fit well in the dichotomy found in this study, between ‘conditions related to severe behavioural disturbance’ and ‘conditions related to sadness and social withdrawal’. In this assessment, only the Burundian respondents had a concept that referred to trauma related complaints. This syndrome, *ihahamuka,* is related to the psychological aftermath of terrible events and is characterized by fear and hyperarousal. This is one of the features of posttraumatic stress disorder. Other features, such as traumatic recollections and avoidance or numbing, were not spontaneously mentioned. Yet, according to Hagengimana and Hinton [45], *guhahamuka* in Rwanda resembles both posttraumatic stress disorder and panic attacks.

The absence of a local category of trauma-related mental disorders in three of the four settings does, of course, not imply that there is no effect of collective violence on the mental state of the population. As has been shown for Juba in South Sudan violent and traumatic events may have pervasive effects on the general physical and mental health of conflict-affected populations [46].

### Limitations

Data yielded by FGDs are often influenced by social dynamics within a group and frequently describe what people assume they should think and do, rather than what people actually think and do. Therefore, our data are limited, and cannot shed light on how the illness categories described here actually play out in people’s lives. Moreover, asking nonaffected people about their observations of affected people may favour mentioning phenomena that are easily observable rather than internal cognitive or emotional states, which are less easily observed by outsiders. Another limitation of this study relates to the role of the researcher. By using local research assistants, who were familiar with the language of the participants, the authors tried to reduce the possibility of interviewer bias. However, the presence of an expatriate researcher in some of the FGDs may have still caused bias. The fact that a non-African representing an international organization providing health services shows interest in the phenomena of ‘madness and sadness’ is in itself a social act with some importance that may have induced social desirability in the responses. Alternatively, participants may also have been reluctant to be honest with someone local in the room.

Finally, there was a limitation with the approach used; through the elicitation of how local syndromes are commonly understood, there is a risk of an ‘essentializing’ approach. This sort of approach focuses on what Nichter [9] calls the ‘whatness’ of particular cultural modes of expressing distress. The authors were aware that local syndromes often have no rigid boundaries, but are used flexibly to interpret illness and misfortune. Yet, this exploratory survey, which identified culturally salient idioms and syndromes, provides a starting point for further, in-depth exploration of how and why specific means of expressing distress, at specific points in time, are being used in concrete situations.

### Conclusions and implications for practice

While cultural categories may be closely aligned to mainstream psychiatric categories, it is important to realize they are not identical and to resist reifying them into professional psychiatric classifications. The local terms used by our respondents are heuristic concepts, used pragmatically to bring order to chaotic and disturbing experiences and to assist in the quests for meaning and solutions to end suffering. These concepts are localized and, therefore, show the influence of contextual factors in shaping illness experience.

This assessment has several implications for public mental health interventions. In the first instance, it clearly shows that the population is concerned about conditions characterized by behavioural disturbances. These conditions share many features with psychotic disorders, as identified by Western psychiatry. People see overwhelmingly the need for these conditions to be treated, but do not know how to do so effectively. Treatment by traditional, or religious, healers is primarily not seen as effective. Neither is treatment within the health care sector an option sought very often, as health facilities do not have staff trained in diagnosis and treatment of mental-health conditions and lack effective medicines for treating these conditions. The population is, however, likely to try any treatment option that seems viable once it is made available to them.

Our conclusions are similar to those of a recent study showing that rural Haitians in areas affected by an earthquake do not seek mental health treatment within the formal health sector as this option is not readily available, and not because they do not wish to try it [47]. Moreover, the evidence for the effectiveness of psychiatric interventions for the management of severe mental disorders, such as psychotic syndromes, is relatively strong [48]. Therefore, we advocate that treatment of severe mental disorders should be made a priority for the health care system. Given the extreme shortage of mental health professionals in low-income countries (approximately one psychiatrist per two million people and one psychiatric nurse per 200,000 people), these interventions cannot be implemented simply by specialists [49]. Evidence suggests, however, that mental health care can be delivered effectively within general health care facilities by nonspecialist health providers, with brief training and appropriate supervision by mental health specialists [50].

Secondly, each population has local categories for states in which a person is overwhelmed by loss or sadness. These conditions are not seen as medical problems or indeed as conditions requiring assistance from the health sector. The interventions considered viable in these instances, by the local population, most often occur within the family and the community. Therefore, the entry point to provide assistance for those who suffer from these conditions would ideally be within the families and the communities. A primary aim for public mental health interventions for these conditions would thus be to empower ‘natural’ social support systems already in place at local levels and to strengthen social cohesion and social capital within communities [51]. However, our respondents also made it clear that the existing mechanisms for healing may fall short or be overwhelmed, particularly in postconflict areas. Local systems of support can be strengthened through capacity building for community-based psychosocial support and by installing services through trained paraprofessional counsellors or community workers [52,53]. It is important that any approach includes various, overlapping levels of interventions in order to address varying needs for support for problems that range from primarily psychosocial to psychiatric [54].

One major challenge to the development of such an integrated, multilevelled care systems among populations that are overwhelmed by massive losses and breakdown of social-support structures is how to determine when mild and/or moderate depressive states become psychiatric conditions requiring medical attention [55,56]. Addressing this problem needs continuous cooperation between health professionals and community resources. Within such a dialogue, it is essential to keep account of how people themselves define what is at stake for their own lives.

## Competing interests

The authors declare that they have no competing interests.

## Authors’ contributions

PV designed the study and data collection tools; oversaw the collection of the survey and focus group data; and wrote the first draft of the paper. MJ reviewed the qualitative data analysis write-up. JJ and RR provided guidance in the analysis and interpretation of results and in the writing of the paper. All authors criticized drafts of the paper, and PV was responsible for subsequent collation of inputs and redrafting. All authors read and approved the final manuscript.
